# Direct Bragg Grating Inscription in Single Mode Step-Index TOPAS/ZEONEX Polymer Optical Fiber Using 520 nm Femtosecond Pulses

**DOI:** 10.3390/polym14071350

**Published:** 2022-03-26

**Authors:** Xuehao Hu, Yuhang Chen, Shixin Gao, Rui Min, Getinet Woyessa, Ole Bang, Hang Qu, Heng Wang, Christophe Caucheteur

**Affiliations:** 1 Research Center for Advanced Optics and Photoelectronics, Department of Physics, College of Science, Shantou University, Shantou 515063, China; 20yhchen6@stu.edu.cn (Y.C.); haqux@stu.edu.cn (H.Q.); 2 Key Laboratory of Intelligent Manufacturing Technology of MOE, Shantou University, Shantou 515063, China; 3 College of Science, Shenyang Aerospace University, Shenyang 110136, China; gaoshixin@stu.sau.edu.cn (S.G.); wangheng@sau.edu.cn (H.W.); 4 Center for Cognition and Neuroergonomics, State Key Laboratory of Cognitive Neuroscience and Learning, Beijing Normal University at Zhuhai, Zhuhai 519087, China; ruimin@bnu.edu.cn; 5 DTU Fotonik, Department of Photonics Engineering, Technical University of Denmark, 2800 Kgs. Lyngby, Denmark; gewoy@fotonik.dtu.dk (G.W.); oban@fotonik.dtu.dk (O.B.); 6 Department of Electromagnetism and Telecommunication, University of Mons, Boulevard Dolez 31, 7000 Mons, Belgium; christophe.caucheteur@umons.ac.be

**Keywords:** fiber Bragg gratings, polymer optical fibers, femtosecond laser, micromachining

## Abstract

We experimentally report fiber Bragg gratings (FBGs) in a single mode step-index polymer optical fiber (POF) with a core made of TOPAS and cladding made of ZEONEX using 520 nm femtosecond pulses and a point-by-point (PbP) inscription method. With different pulse energies between 9.7 nJ and 11.2 nJ, 12 FBGs are distributed along the cores of two pieces of POFs with negative averaged effective index change up to ~6 × 10^−4^ in the TOPAS. For POF 1 with FBGs 1–6, the highest reflectivity 45.1% is obtained with a pulse energy of 10.6 nJ. After inscription, good grating stability is reported. Thanks to the post-annealing at 125 °C for 24 h, after cooling the grating reflectivity increases by ~10%. For POF 2 with FBGs 7–12, similar FBG data are obtained showing good reproducibility. Then, the FBGs are annealed at 125 °C for 78 h, and the average reflectivity of the FBGs during the annealing process increases by ~50% compared to that before the annealing, which could be potentially applied to humidity insensitive high temperature measurement.

## 1. Introduction

Fiber Bragg gratings (FBGs) are very critical fiber-optic components widely used not only in telecommunication but also in sensing applications [1]. Since the first FBG was fabricated on a silica optical fiber by Hill et al. in 1978 [2], UV lasers and phase masks have been the most popular devices for FBG manufacturing. However, driven by the demands for novel FBGs able to operate in high temperature environments (400 °C to 1800 °C) or used as vector bending sensors, femtosecond lasers are widely becoming extremely useful because of ultra-short pulse width and extreme-high peak power, which can induce fiber core refractive index change in diverse transparent materials, such as silica, single crystal, glasses, etc., [3]. Compared to phase mask technology and holographic interferometry, direct writing technologies, such as point-by-point (PbP) and line-by-line (LbL) for FBG inscriptions, demonstrate unique advantages of high accuracy with reduced thermal effect, since refractive indices could be modified to the size of sub-microns inside transparent materials by focusing a femtosecond laser beam by a high NA objective with high magnification [4,5,6,7].

In addition to FBGs in silica fibers, FBGs in polymer optical fibers (POFs) have been more and more popular since the first fabrication in 1999 [8] due to their unique advantages compared to silica fibers, such as lower Young’s modulus, larger negative thermo-optic coefficients, higher elastic strain limits, and higher bending flexibility [9,10,11]. FBGs in POFs have been used for a variety of sensing applications, such as temperature [12,13], strain [14], humidity [15,16], liquid level [17], plantar pressure [18], gas pressure [19] and soil water content [20]. Meanwhile, researchers have tried to improve FBG reflectivity and inscription efficiency by different techniques. In 2017, Pospori et al. inscribed an FBG a benzyl dimethyl ketal (BDK)-doped poly(methyl methacrylate) (PMMA) microstructured POFs with a reflectivity of 98.4% using a single 248 nm KrF excimer laser pulse and phase mask technique [21]. Compared to femtosecond lasers, this UV pulsed laser is cheaper. Additionally, FBG production in POFs with a femtosecond laser and the phase mask technique were also reported. In 2017, Hu et al. produced FBGs in single mode BDK-doped PMMA microstructured POFs by means of a 400 nm femtosecond pulsed laser, showing an FBG reflectivity of 83% with inscription time of only 40 s [22].

Though the femtosecond laser and phase mask technology have been used to fabricate highly reflective FBGs in POFs, the production of FBGs with different Bragg wavelengths requires the use of different phase masks. Therefore, femtosecond laser direct writing techniques, such as PbP, LbL and Plane-by-Plane (Pl-b-Pl) were proposed and developed to gain flexibility. In 2012, Stefani et al. reported PbP laser direct writing of a fourth-order FBG with a reflection peak of ~10 dB at 1520 nm for the first time in a microstructured PMMA POF. The specially designed microstructure facilitated the propagation of the writing beam [23]. Since 2015, the researchers at Cyprus University of Technology inscribed fourth-order FBGs of reflectivity ~70% in CYTOP perfluorinated POFs by the LbL [24] or Pl-b-Pl [25] method with a femtosecond laser operating at 517 nm. In 2021, Dash et al. drew a novel step-index rectangular cyclic olefin polymer ZEONEX POFs and then fourth and higher-order FBGs were obtained using a 520 nm femtosecond laser and the PbP technique. Three reflection peaks were exhibited at 866.8 nm, 1013 nm and 1511.3 nm [26].

Compared to a fourth-order grating, for a given grating length and refractive index modulation amplitude, the quality of the FBG with a first-order grating structure is superior, since for the latter the number of the grating period is reduced. On the other hand, to obtain the desired grating reflectivity the grating length and pulse energy can be reduced significantly with a first-order grating structure [27]. Recently, we reported first-order gratings in step-index BDK-doped PMMA POFs by the PbP technique with a reflectivity ~99% [28]. However, the BDK could bring extra losses for the POFs and the operational temperature for PMMA-based gratings is limited to ~80 °C. Thus, in this work, we aim to develop first-order FBGs in step-index TOPAS-ZEONEX POFs without dopants for the first time to the best of our knowledge and then investigate the stability of the FBGs at a higher temperature up to 125 °C.

We demonstrate 12 first-order FBGs in two pieces (six FBGs for POF 1 and another six FBGs for POF 2) of single mode step-index POFs with core material TOPAS and cladding material ZEONEX. Two pieces of POFs (POF 1 and POF 2) are identical for the investigation of FBG reproducibility. By variations of pulse energies of the femtosecond laser emitting at 520 nm, 12 FBGs (2 mm in length) are achieved. After FBG inscriptions, grating stabilities at room temperature and at 125 °C for up to 78 h are investigated.

## 2. Experimental Set-Up

The single mode step-index POF used in this work was fabricated in-house at DTU Fotonik. The core and cladding of the fiber are made of TOPAS (5013S-04, TOPAS Advanced Polymers, Raunheim, Germany) and ZEONEX (480R, ZEON CORPORATION, Tokyo, Japan), respectively. Their refractive indices are 1.5238 and 1.5179, respectively, at 850 nm. Both materials are humidity insensitive, and they can operate over 130 °C, as the glass transition temperature ( $T_{g}$ ) of these materials is 138 °C and 134 °C, respectively. Injection molding was used to fabricate the step index preform, and then the fiber was fabricated by a fiber drawing tower with a core and cladding diameter of 5 μm and 128 μm, respectively. More information about this fiber can be found in the work conducted by Woyessa et al. [29]. Based on the property of the fiber materials, the FBGs inscribed in the POF could be applied to humidity insensitive high temperature measurement.

**Figure 1:**
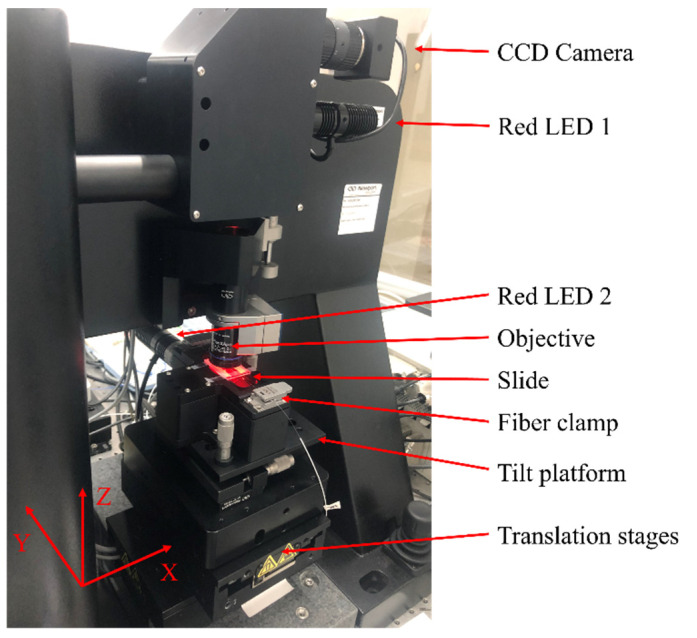
Illustration of PbP FBG inscription set-up.

FBG inscriptions were performed by a laser micromachining system from Newport Corporation, Irvine, CA, United States, including a 520 nm femtosecond laser (SpOne-8-SHG), which features pulse duration of 306 fs, repetition rate up to 200 kHz and maximum pulse energy of 28.2 μJ, three-axis precision translation stages (X/Y: XMS100-S, Z: M-VP-5ZA, from bottom to top), and a corresponding micromachining platform with customized software. The linear-polarized beam emitted from the laser output, in turn, goes through a tunable half-waveplate, a Glan laser polarizer, a collimator, and a quarter-wave plate, generating variable energies and circularly polarized pulses to significantly reduce the anisotropy of refractive index change [30]. Then the laser beam is focused onto the fiber by an oil-immersion objective (60×, NA = 1.42, UPLXAPO60XO, OLYMPUS, Shinjuku City, Japan). The diameter of the focused beam spot is 447 nm according to the diffraction limit D = 1.22λ/NA [31]. LED illumination and CCD real-time monitoring system are integrated into this system to facilitate the FBG inscription. The FBG inscription setup is illustrated in Figure 1.

To reduce the large transmission loss of the POFs, small pieces of the POFs (~5 cm long) were adopted for the experiment. Before FBG inscription, they were pre-annealed at 130 °C for two days for the release of frozen-in stress generated during the fiber drawing process [29]. Then, they were connected to silica fiber pigtails by UV-glue (Norland 86H) in order to monitor the spectra during the grating inscription process. Then, the pre-strained POF was mounted on a glass slide by two pieces of tape. The fiber alignment was conducted with the help of the multi-axis tilt platform (M-37, Newport) positioned above the top precision translation stage. During FBG inscription, the fiber was translated along the Y-axis, and hence periodical refractive index modulation on the fiber core was induced by the focused femtosecond laser pulses. By adjusting the translation velocity v and/or the pulse repetition rate f of the laser, the period Λ of the FBG can be controlled freely by:

(1) Λ=v/f

The grating spectra were recorded by an interrogator (FS22SI Industrial BraggMETER Interrogator) from HBM FiberSensing, Moreira, Portugal with a wavelength resolution of 1 pm and a scanning rate of 1 Hz. With the use of a fiber circulator, both reflected and transmitted spectra were obtained.

## 3. Experimental Results and Discussion

### 3.1. FBG Inscription Using 520 nm Femtosecond Pulses

In this work, 2-mm-long FBGs were fabricated in two pieces of POFs using the femtosecond laser PbP inscription method. It is worth mentioning that longer FBGs could be inscribed using this technique. However, in this work, we focus on the investigation of the refractive index change mechanism and high temperature stabilities of the FBGs. f was set to a constant value of 50 Hz, while v was varied from 24.5 μm/s to 25.95 μm/s to generate distributed FBGs at different Bragg wavelengths $\lambda_{B}$ with an interval of 1 mm. Meanwhile, the pulse energy E and fluence F were increased from 9.7 nJ to 11.2 nJ and from 6.2 J/cm^2^ to 7.2 J/cm^2^, respectively, to compare the FBG inscription performances (see Table 1).

**Figure 2:**
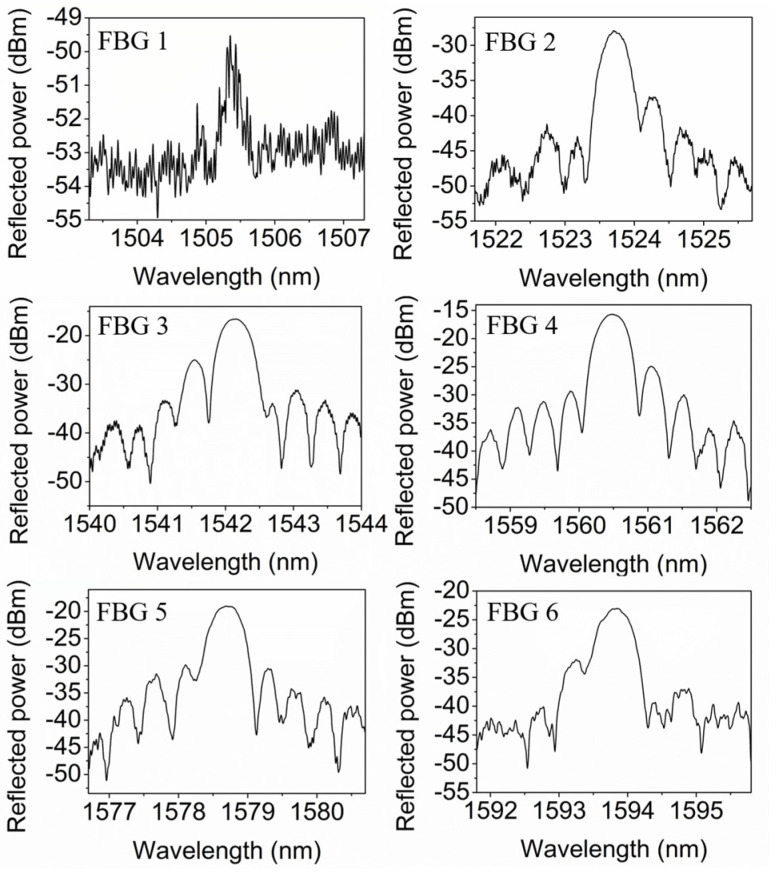
Reflected spectra of distributed 2-mm-long FBGs 1–6 in POF 1 inscribed with an interval of 1 mm by the femtosecond laser at 520 nm.

Figure 2 presents the reflected spectra for FBGs 1–6 in POF 1 in the range from 1500 nm to 1600 nm. The full width half maximum of the peak is ~0.4 nm for FBGs 2–6. The large 3-dB width could be due to the short grating length of 2 mm. The effective index of the FBG $n_{eff,FBG}$ is a sum of the effective index of the single mode fiber without the FBG $n_{eff}$ and the averaged effective index change due to the FBG $\bar{\delta n_{eff}}$ . Because of the extremely small reflectivity, no sign of FBG 1 is observed in transmission, thus $\bar{\delta n_{eff}}$ is omitted. $\lambda_{B}$ was obtained based on the FBG spectrum just after inscription with the pre-strain. Thus, $n_{eff}$ is estimated to be 1.53608 by [32]:

(2) $$\lambda_{B}=2\left(n_{eff}+\bar{\delta n_{eff}}\right)\Lambda$$

**Figure 3:**
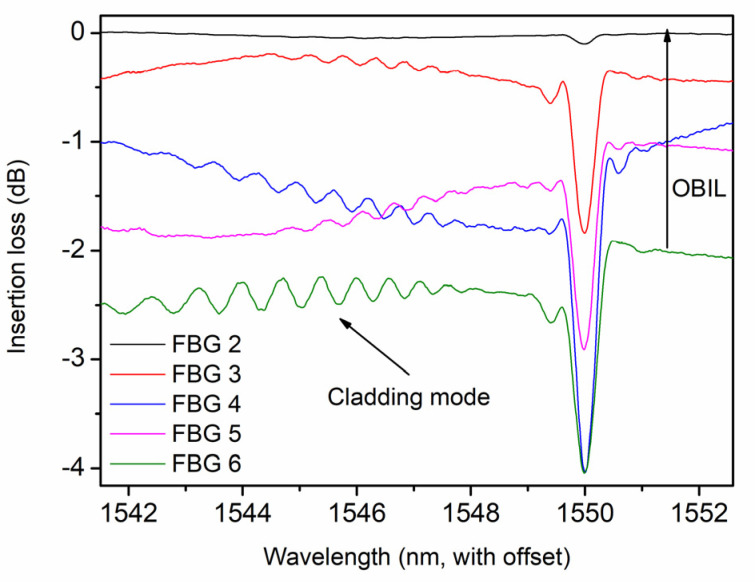
Insertion losses of FBGs 2–6 in POF 1 due to inscriptions by the femtosecond laser at 520 nm.

For FBGs 2–6, the insertion losses induced by laser pulses are depicted in Figure 3. The cladding modes to the left of the core modes are presented similarly as FBGs inscribed by He et al. in silica optical fibers using the PbP method, which could be attributed to the inhomogeneity of the refractive index modulation in a cross-section of fiber induced by femtosecond laser direct writing technology [3] and the cladding modes could be reduced by using POFs with thinner cores. With increasing pulse energies, the reflectivity of FBG 3 increases to 28.2% with an out-of-band insertion loss (OBIL) of 0.350 dB, and the counterpart of FBG 4 improves to 45.1% with a pulse energy of 10.6 nJ, which is the maximum among all gratings. However, the OBIL due to laser irradiation increases to 1.082 dB. Finally, for FBG 6 with the highest pulse energy of 11.2 nJ, the reflective decreases to 34.3% with a maximal OBIL of 1.919 dB. Thus, there is a tradeoff between reflectivity and the OBIL.

According to Equation (2), negative $\bar{\delta n_{eff}}$ due to laser irradiation was obtained, and the absolute value increases from 0.00009 to 0.00059. The negative index change could be attributed to the main chain scission caused by laser irradiation, which leads to shorter chain molecules with a higher degree of freedom and lower density. The negative index change property also applies to PMMA/Polystyrene copolymer irradiated by the 308 nm excimer laser [33] but is different from the counterpart in PMMA material which normally has a positive index change with femtosecond irradiation [34,35]. The maximal fringe visibility *γ* corresponding to the amplitude of the effective index change is equal to 1.38, belonging to FBG 3, calculated by:

(3) $$R=tanh^{2}\left(\frac{\pi }{\lambda_{B}}\gamma \bar{\delta n_{eff}}L\right),$$

where R and L represent the grating reflectivity and the grating length, respectively. The value *γ* for FBG 3 larger than 1 could be attributed to the refractive index increase in the non-irradiated area. More FBG data for POF 1 are presented in Table 1.

**Figure 4:**
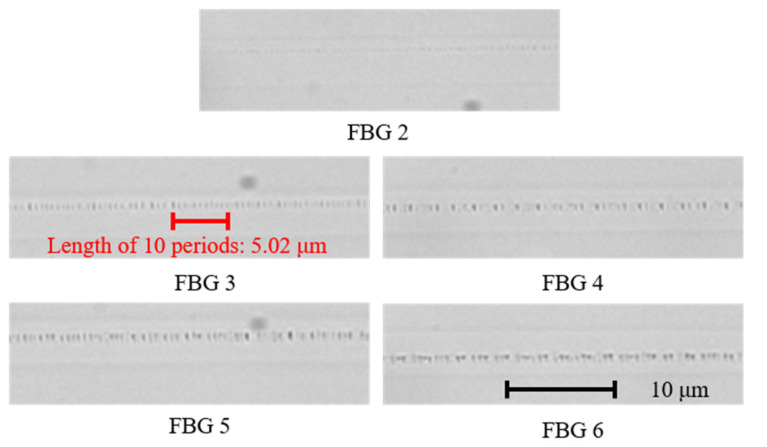
Optical microscope images of distributed FBGs 2–6 in POF 1 inscribed by the femtosecond laser at 520 nm.

Figure 4 depicts a high-resolution microscope image of FBGs 2–6 using the microscope (SAGA, SJ-U500) with an oil-immersion objective (100×, NA = 1.25). It is worth mentioning that no refractive index modifications are observed in FBG 1 due to the low pulse energy, despite of the presence of the small grating peak. For FBG 2, very weak modifications are displayed. For FBG 3, modifications induced by laser pulses are observed with an estimated length of ~300 nm along the fiber axis. This index modification in small size could be attributed to highly nonlinear defect formation resulting from a multi-photon absorption process, and thus the size of the index modification region could be much smaller than that of the focused femtosecond laser beam [36]. With even higher pulse energies, the modifications appear to be irregular, which may be the reason for the increase of the OBIL, as shown in Figure 3. Thus, we assume that with E smaller than 9.7 nJ, no FBG could be obtained, and with E larger than 11.2 nJ, POFs could be seriously damaged.

Additionally, FBGs 7–12 in POF 2 were inscribed using the same methodology with similar parameters as FBGs 1–6 in POF 1, confirming the reproducibility of the FBGs in the POFs using the femtosecond laser direct writing system. The full FBG data for POF 2 are presented in Table 1.

### 3.2. FBG Stability Analysis

**Figure 5:**
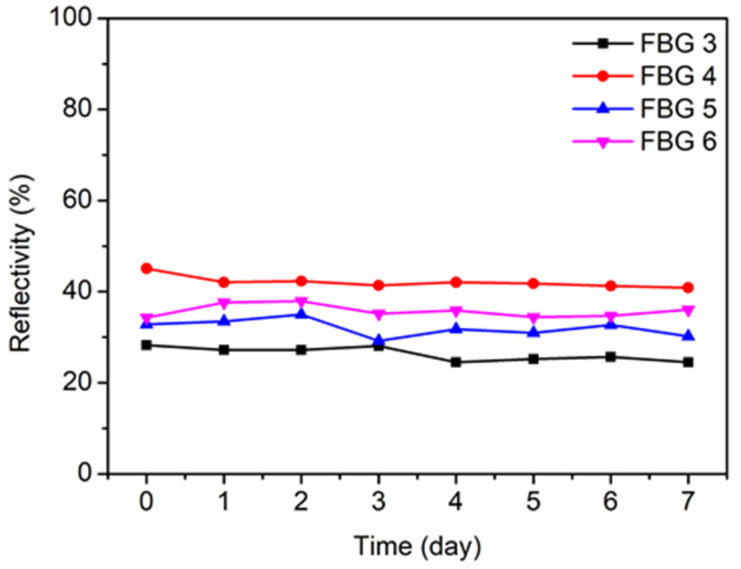
The reflectivity evolutions of FBGs 3–6 in POF 1 during the 7 days after inscriptions.

For FBGs 1–6 in POF 1, after the inscriptions, their stabilities were investigated by recording both the transmitted and reflected amplitude spectra for 7 days. Here, the reflectivity calculated from the transmitted spectra is considered to better reflect the evolutions of the grating quality. Though there are six reflected spectra for the FBGs, only the transmitted spectra of FBGs 3–6 are observable, as shown in Figure 5. The time 0 in the horizontal axis represents the moment just after inscription. The reflectivity of the FBG almost remained the same value showing stable property of the FBG inscribed by the high peak power density of the femtosecond laser.

**Figure 6:**
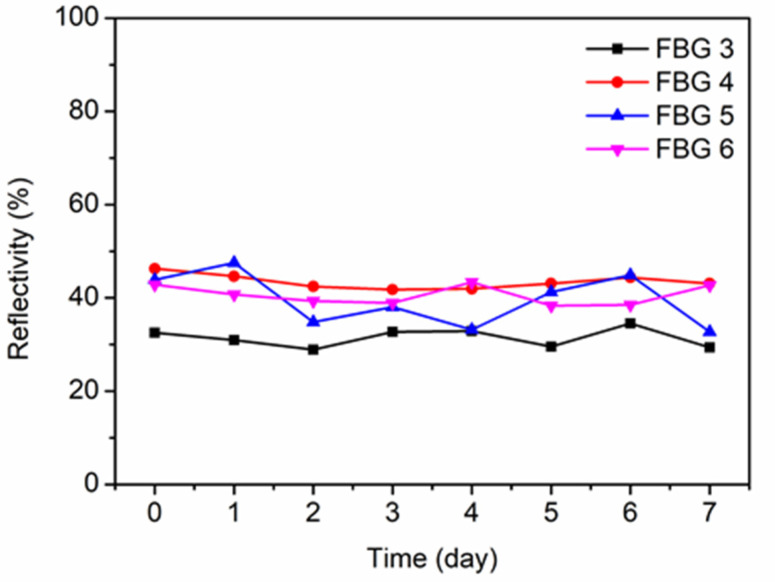
The reflectivity evolutions of FBGs 3–6 in POF 1 during the 7 days after post-annealing at 125 °C for 24 h.

**Figure 7:**
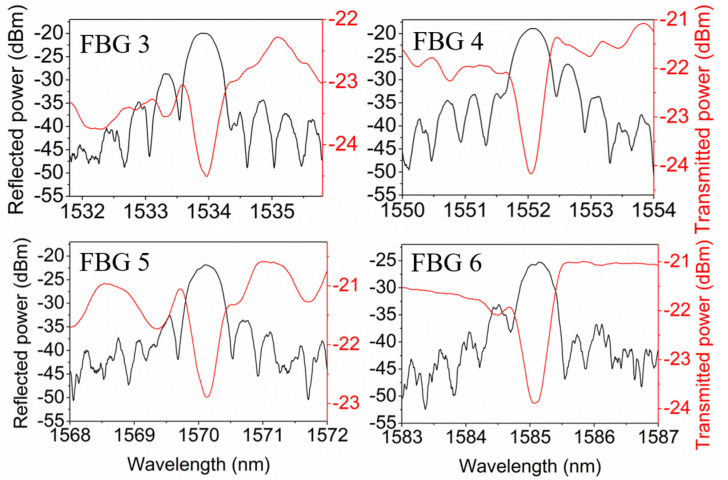
Reflected and transmitted amplitude spectra of 2-mm-long FBGs 3–6 in POF 1 in day 7 after fiber cooling.

Afterward, POF 1 was post-annealed for 24 h, as reported in [29]. The highest post-annealing temperature was set to 125 °C higher than 110 °C for the same fiber reported in [29] because the cured UV-glue can only withstand temperatures from −125 °C to 125 °C. After that, FBGs 3–6 were monitored at room temperature for another 7 days, as depicted in Figure 6. The time 0 in the horizontal axis represents the moment when the fiber was cooled down to room temperature after post-annealing. Averagely, the reflectivity of the FBGs increased by ~10% due to the post-annealing, though the reflectivity fluctuations existed. Both reflected and transmitted amplitude spectra in day 7 are depicted in Figure 7 showing good grating qualities. Despite the appearance of the side-lobes, it is easy to interrogate the main reflection peak by commercial software. Nevertheless, the side-lobes can be reduced by means of the apodization method using variable pulse energies.

**Figure 8:**
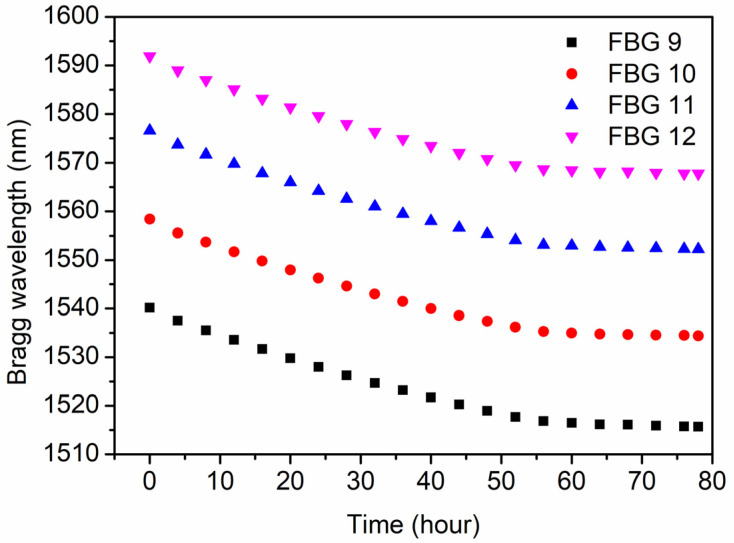
The Bragg wavelength evolutions of FBGs 9–12 in POF 2 during the annealing process at 125 °C for 78 h.

**Figure 9:**
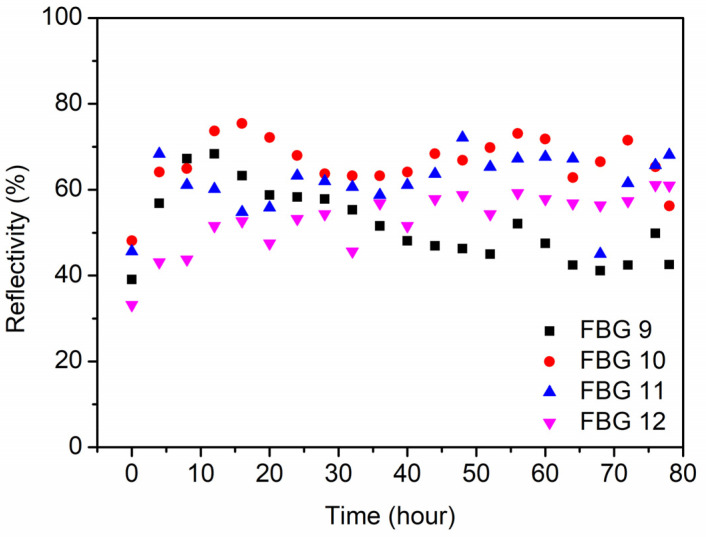
The reflectivity evolutions of FBGs 9–12 in POF 2 during the annealing process at 125 °C for 78 h.

For FBGs 9–12 in POF 2, long term temperature stability at 125 °C was investigated, and the dynamic wavelength shift and reflectivity were recorded according to the evolution of the spectra for 78 h, as depicted in Figure 8 and Figure 9, respectively. The time 0 in the horizontal axis represents the moment at room temperature. It is found that the Bragg wavelength of FBGs 9–12 obviously blue-shifted during the first 60 h of the post-annealing process, which could be caused by the further release of the frozen-in stress and the negative thermo-optic coefficient of PMMA [37]. Afterward, the Bragg wavelength almost stopped shifting. Meanwhile, during the post-annealing process, the reflectivity of the FBG fluctuated at ~60%, and the average reflectivity of the FBGs increased by ~50% compared to the time 0 at room temperature.

**Figure 10:**
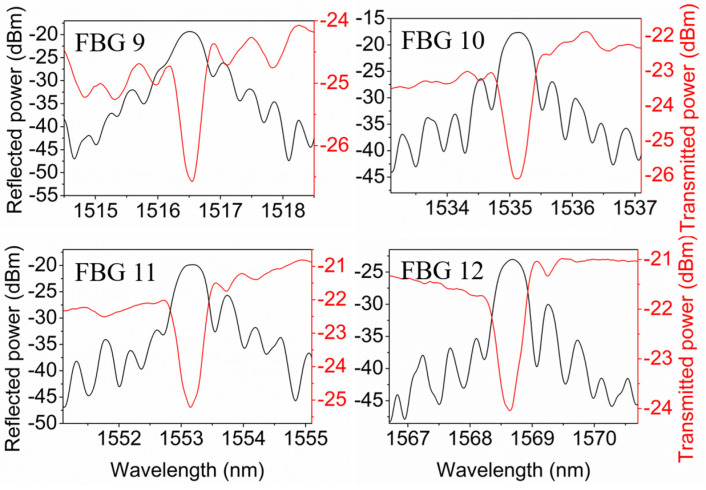
Reflected and transmitted amplitude spectra of 2-mm-long FBGs 9–12 in POF 2 at room temperature after the post-annealing at 125 °C for 78 h.

After the post-annealing at 125 °C for 78 h, the spectra of FBGs 9–12 at room temperature were recorded, as shown in Figure 10, showing a Bragg wavelength blue shift of ~23 nm compared to the Bragg wavelength just before the post-annealing. The reflectivities of FBGs 9–12 were 37.2%, 53.3%, 54.9% and 6.4%, respectively, with good grating shapes.

It is worth mentioning that femtosecond laser induced gratings have a long term regeneration effect due to the side chain and backbone relaxation processes in polymers. In addition, annealing close to $T_{g}$ temperature accelerates regeneration, influencing the long term stability of inscribed gratings in polymers. This phenomenon is even observed in inorganic glasses [38]. For our FBGs 3–6 and FBGs 9–12, the reflectivity remained almost the same after 6 months and 5 months, respectively, from the post-annealing process, showing good long-term stability.

## 4. Conclusions

In this work, we have studied first-order FBGs inscribed in single mode step-index POFs with a core made of TOPAS and cladding made of ZEONEX by a femtosecond laser and point-by-point technique. Overall, 12 FBGs (2 mm in length) were obtained with different pulse energies between 9.7 nJ and 11.2 nJ in two pieces of POFs with a negative averaged effective index change of up to ~6 × 10^−4^ in TOPAS. For POF 1 with FBGs 1–6, the highest reflectivity, 45.1%, was obtained with a pulse energy of 10.6 nJ. Thanks to the post-annealing at 125 °C for 24 h, after cooling the grating reflectivity increased by ~10%. For POF 2 with FBGs 7–12, similar FBG inscription results were obtained. Afterward, the FBGs were post-annealed at 125 °C for 78 h to demonstrate long-term temperature stability, and the average reflectivity of the FBGs during the annealing process increased by ~50% compared to the time 0 at room temperature. Thus, FBGs could be potentially applied to humidity insensitive high temperature measurement.

## Figures and Tables

**Table 1:** Data of distributed FBGs inscribed by the femtosecond laser at 520 nm.

	POF 1						POF 2					
FBG	1	2	3	4	5	6	7	8	9	10	11	12
E (nJ)	9.7	10.0	10.3	10.6	10.9	11.2	9.7	10.0	10.3	10.6	10.9	11.2
F (J/cm^2^)	6.2	6.4	6.6	6.8	7.0	7.2	6.2	6.4	6.6	6.8	7.0	7.2
v (μm/s)	24.5	24.8	25.1	25.4	25.7	25.95	24.5	24.8	25.1	25.4	25.7	25.95
Λ (μm)	0.490	0.496	0.502	0.508	0.514	0.519	0.490	0.496	0.502	0.508	0.514	0.519
$\lambda_{B}$ (nm)	1505.36	1523.70	1542.12	1560.46	1578.69	1593.84	1505.83	1524.32	1542.42	1560.69	1578.93	1594.30
$n_{eff,FBG}$	1.53608	1.53599	1.53597	1.53589	1.53569	1.53549	1.53656	1.53661	1.53627	1.53611	1.53592	1.53593
$\bar{\delta n_{eff}}$	~0	−0.00009	−0.00011	−0.00019	−0.00039	−0.00059	~0	0.00005	−0.00029	−0.00045	−0.00064	−0.00063
OBIL (dB)	~0	0.018	0.350	1.082	1.011	1.919	~0	0.141	0.034	0.902	1.523	1.880
R (%)	~0	2.0	28.2	45.1	32.8	34.3	~0	12.9	43.5	48.3	52.8	30.9
γ	~0	0.39	1.38	1.06	0.42	0.29	~0	1.77	0.68	0.47	0.36	0.25
