# Correction: Hu et al. Direct Bragg Grating Inscription in Single Mode Step-Index TOPAS/ZEONEX Polymer Optical Fiber Using 520 nm Femtosecond Pulses. *Polymers* 2022, *14*, 1350

**DOI:** 10.3390/polym14132640

**Published:** 2022-06-29

**Authors:** Xuehao Hu, Yuhang Chen, Shixin Gao, Rui Min, Getinet Woyessa, Ole Bang, Hang Qu, Heng Wang, Christophe Caucheteur

**Affiliations:** 1 Research Center for Advanced Optics and Photoelectronics, Department of Physics, College of Science, Shantou University, Shantou 515063, China; 20yhchen6@stu.edu.cn (Y.C.); haqux@stu.edu.cn (H.Q.); 2 Key Laboratory of Intelligent Manufacturing Technology of MOE, Shantou University, Shantou 515063, China; 3 College of Science, Shenyang Aerospace University, Shenyang 110136, China; gaoshixin@stu.sau.edu.cn (S.G.); wangheng@sau.edu.cn (H.W.); 4 Center for Cognition and Neuroergonomics, State Key Laboratory of Cognitive Neuroscience and Learning, Beijing Normal University at Zhuhai, Zhuhai 519087, China; ruimin@bnu.edu.cn; 5 DTU Fotonik, Department of Photonics Engineering, Technical University of Denmark, 2800 Kgs. Lyngby, Denmark; gewoy@fotonik.dtu.dk (G.W.); oban@fotonik.dtu.dk (O.B.); 6 Department of Electromagnetism and Telecommunication, University of Mons, Boulevard Dolez 31, 7000 Mons, Belgium; christophe.caucheteur@umons.ac.be

## Error in Table

The authors wish to make a change to the published paper [1]. In the original publication, there was a mistake in Table 1 as published. “**The laser pulse energy and pulse fluence applied on the fiber during grating fabrication were not correct**”. The corrected rows 3, 4 of Table 1 appears below.



**POF 1**

**POF 2**

**FBG**

**1**

**2**

**3**

**4**

**5**

**6**

**7**

**8**

**9**

**10**

**11**

**12**
E (nJ)9.710.010.310.610.911.29.710.010.310.610.911.2F (J/cm^2^)6.26.46.66.87.07.26.26.46.66.87.07.2

## Text Correction

There was an error in the original publication. “**The laser pulse energy and pulse fluence applied on the fiber during grating fabrication were not correct**”.

A correction has been made to “***Abstract***”:

“**Abstract:** We experimentally report fiber Bragg gratings (FBGs) in a single mode step-index polymer optical fiber (POF) with a core made of TOPAS and cladding made of ZEONEX using 520 nm femtosecond pulses and a point-by-point (PbP) inscription method. With different pulse energies between 9.7 nJ and 11.2 nJ, 12 FBGs are distributed along the cores of two pieces of POFs with negative averaged effective index change up to ~6 × 10^−4^ in the TOPAS. For POF 1 with FBGs 1–6, the highest reflectivity 45.1% is obtained with a pulse energy of 10.6 nJ. After inscription, good grating stability is reported. Thanks to the post-annealing at 125 °C for 24 h, after cooling the grating reflectivity increases by ~10%. For POF 2 with FBGs 7–12, similar FBG data are obtained showing good reproducibility. Then, the FBGs are annealed at 125 °C for 78 h, and the average reflectivity of the FBGs during the annealing process increases by ~50% compared to that before the annealing, which could be potentially applied to humidity insensitive high temperature measurement.”

A correction has been made to “***3. Experimental Results and Discussion, 3.1. FBG Inscription Using 520 nm Femtosecond Pulses, Paragraph 1***”:

“In this work, 2-mm-long FBGs were fabricated in two pieces of POFs using the femtosecond laser PbP inscription method. It is worth mentioning that longer FBGs could be inscribed using this technique. However, in this work, we focus on the investigation of the refractive index change mechanism and high-temperature stabilities of the FBGs. f was set to a constant value of 50 Hz, while v was varied from 24.5 μm/s to 25.95 μm/s to generate distributed FBGs at different Bragg wavelengths $\lambda_{B}$ with an interval of 1 mm. Meanwhile, the pulse energy E and fluence F were increased from 9.7 nJ to 11.2 nJ and from 6.2 J/cm^2^ to 7.2 J/cm^2^, respectively, to compare the FBG inscription performances (see Table 1).”

A correction has been made to “***3. Experimental Results and Discussion, 3.1. FBG Inscription Using 520 nm Femtosecond Pulses, Paragraph 3***”:

“For FBGs 2–6, the insertion losses induced by laser pulses are depicted in Figure 3. The cladding modes to the left of the core modes are presented similarly as FBGs inscribed by He et al. in silica optical fibers using the PbP method, which could be attributed to the inhomogeneity of the refractive index modulation in a cross-section of fiber induced by femtosecond laser direct writing technology [3] and the cladding modes could be reduced by using POFs with thinner cores. With increasing pulse energies, the reflectivity of FBG 3 increases to 28.2% with an out-of-band insertion loss (OBIL) of 0.350 dB, and the counterpart of FBG 4 improves to 45.1% with a pulse energy of 10.6 nJ, which is the maximum among all gratings. However, the OBIL due to laser irradiation increases to 1.082 dB. Finally, for FBG 6 with the highest pulse energy of 11.2 nJ, the reflective decreases to 34.3% with a maximal OBIL of 1.919 dB. Thus, there is a tradeoff between reflectivity and the OBIL.”

A correction has been made to “***3. Experimental Results and Discussion, 3.1. FBG Inscription Using 520 nm Femtosecond Pulses, Paragraph 5***”:

“Figure 4 depicts a high-resolution microscope image of FBGs 2–6 using the microscope (SAGA, SJ-U500) with an oil-immersion objective (100×, NA = 1.25). It is worth mentioning that no refractive index modifications are observed in FBG 1 due to the low pulse energy, despite the presence of the small grating peak. For FBG 2, very weak modifications are displayed. For FBG 3, modifications induced by laser pulses are observed with an estimated length of ~300 nm along the fiber axis. This index modification in small size could be attributed to highly nonlinear defect formation resulting from a multiphoton absorption process; thus, the size of the index modification region could be much smaller than that of the focused femtosecond laser beam [36]. With even higher pulse energies, the modifications appear to be irregular, which may be the reason for the increase in the OBIL, as shown in Figure 3. Thus, we assume that with E smaller than 9.7 nJ, no FBG could be obtained, and with E larger than 11.2 nJ, POFs could be seriously damaged.”

A correction has been made to “***4. Conclusions***”:

“In this work, we have studied first-order FBGs inscribed in single-mode step-index POFs with a core made of TOPAS and cladding made of ZEONEX by a femtosecond laser and point-by-point technique. Overall, 12 FBGs (2 mm in length) were obtained with different pulse energies between 9.7 nJ and 11.2 nJ in two pieces of POFs with a negative averaged effective index change of up to ~6 × 10^−4^ in TOPAS. For POF 1 with FBGs 1–6, the highest reflectivity, 45.1%, was obtained with a pulse energy of 10.6 nJ. Thanks to the post-annealing at 125 °C for 24 h, after cooling the grating reflectivity increased by ~10%. For POF 2 with FBGs 7–12, similar FBG inscription results were obtained. Afterward, the FBGs were post-annealed at 125 °C for 78 h to demonstrate long-term temperature stability, and the average reflectivity of the FBGs during the annealing process increased by ~50% compared to the time 0 at room temperature. Thus, FBGs could be potentially applied to humidity-insensitive high-temperature measurement.”

The authors apologize for any inconvenience caused and state that the scientific conclusions are unaffected. This correction was approved by the Academic Editor. The original publication has also been updated.
